# A Simplified CAMBRA-Based Diagnostic Caries Risk Assessment Tool for Young Adults: Development and Clinical Validation

**DOI:** 10.3390/diagnostics16060859

**Published:** 2026-03-13

**Authors:** Liana Beresescu, Alexandra Mihaela Stoica, Andrea Bors, Alina Ormenisan, Gabriela Felicia Beresescu, Andreea Lucaciu, Elena Stepco, Csilla Benedek

**Affiliations:** 1 Faculty of Dental Medicine, University of Medicine and Pharmacy, Science, and Technology George Emil Palade, 540139 Târgu-Mureș, Romania; liana.beresescu@umfst.ro (L.B.); andrea.bors@umfst.ro (A.B.); alina.ormenisan@umfst.ro (A.O.); gabriela.beresescu@umfst.ro (G.F.B.);; 2 Faculty of Dental Medicine, The State University of Medicine and Pharmacy “Nicolae Testemitanu”, 2004 Chisinau, Moldova

**Keywords:** caries risk assessment, CAMBRA, young adults, preventive dentistry

## Abstract

**Background/Objectives**: Young adulthood is a transitional period associated with changes in lifestyle and preventive dental attendance, which may influence caries risk. In routine practice, the use of comprehensive caries risk assessment systems is often limited by time and diagnostic requirements, highlighting the need for simplified diagnostic screening tools. This study aimed to develop and clinically validate a simplified, questionnaire-based caries risk assessment tool derived from the CAMBRA framework for use in young adults. **Methods**: An observational cross-sectional study was conducted among 246 Romanian young adults aged 18–25 years. The instrument was designed to enable rapid caries risk stratification based exclusively on questionnaire data, without radiographic or laboratory investigations. Internal consistency, test–retest reliability, and construct validity were evaluated by comparison with clinically recorded indicators, including DMFT values, early enamel changes, visible dental plaque, and active carious lesions. **Results**: The questionnaire showed acceptable internal consistency (Cronbach’s alpha = 0.71) and good temporal stability (ICC = 0.82). Higher caries risk categories were consistently associated with unfavorable clinical findings, including increased DMFT values, a higher prevalence of early enamel changes, greater plaque accumulation, and more frequent active caries (*p* < 0.01). **Conclusions**: The simplified CAMBRA-based questionnaire demonstrated satisfactory reliability and clinical relevance in young adults. It may serve as a practical diagnostic screening and decision-support tool for risk-based caries prevention in routine and community dental settings.

## 1. Introduction

In routine dental practice, caries remains a common finding across the lifespan. Despite the widespread availability of preventive information, preventive measures are not consistently applied in all settings, and clinical examination frequently reveals new carious lesions, early enamel changes, or signs of ongoing disease activity. Epidemiological data consistently show that dental caries remains highly prevalent across age groups, with a substantial proportion of lesions remaining untreated despite the availability of preventive strategies [1,2,3,4,5].

Against this background, caries risk assessment has assumed a central role in everyday caries management. The recognition that caries is a dynamic, multifactorial disease, with risk profiles varying substantially between individuals, has supported a broader shift from lesion-centered treatment toward risk-based prevention and individualized care planning [6]. Within this framework, the Caries Management by Risk Assessment (CAMBRA) approach integrates biological, behavioral, and protective factors into a structured assessment process and is widely used in clinical practice [7,8]. Nevertheless, the routine implementation of comprehensive CAMBRA-based protocols may be challenging in some settings due to time constraints and the need for clinical, radiographic, or laboratory-based information.

Within this broader risk-based framework, young adulthood represents a transitional stage in terms of caries risk and preventive needs. During this period, changes in lifestyle, dietary habits, oral hygiene routines, and dental attendance are common and may increase susceptibility to caries. At the same time, regular dental visits often become less frequent after adolescence, and preventive interventions may be inconsistently applied [9,10]. In this context, early identification of individuals at increased caries risk becomes essential to allow timely preventive measures and to limit progression toward cavitated lesions requiring restorative or endodontic treatment.

In everyday preventive dental practice, particularly in community or population-based contexts, clinicians often require simplified and rapid tools that allow initial caries risk stratification without extensive diagnostic procedures. As highlighted by Featherstone et al., existing caries risk assessment systems differ substantially in terms of structure, complexity, and data requirements, which may limit their routine application in real-world clinical settings [11]. Questionnaire-based instruments represent a pragmatic alternative for this purpose, as they rely on self-reported information related to established caries risk indicators, including dietary habits, oral hygiene behaviors, fluoride exposure, and relevant medical factors. When appropriately designed and applied, such tools may facilitate large-scale screening, support prioritization of preventive resources, and assist clinical decision-making, particularly in settings where comprehensive clinical assessment is not immediately feasible [12,13].

Several caries risk assessment systems have been described in the literature, including comprehensive models based on the CAMBRA framework and other multifactorial risk assessment approaches [7,11]. However, many of these approaches rely on detailed clinical, radiographic, or biological information, which may limit their use as rapid screening tools in routine preventive settings. Although questionnaire-based approaches have been explored in epidemiological research [12,13], simplified instruments specifically designed for rapid caries risk screening in young adult populations remain relatively limited. Within this context, the development of practical tools that allow initial risk stratification using easily obtainable information may be clinically useful.

Simplified questionnaire-based instruments may serve as practical diagnostic screening tools, enabling early identification of individuals at increased caries risk and supporting risk-based preventive decision-making in routine dental practice.

The clinical usefulness of simplified caries risk questionnaires, however, depends on their psychometric robustness and their ability to reflect actual disease patterns. Validation against clinical indicators of caries experience and activity is therefore essential to ensure that questionnaire-derived risk scores correspond to meaningful differences in oral health status [14,15,16]. Demonstrating coherent associations with parameters such as DMFT values, early enamel changes, visible dental plaque, and active cavitated lesions provides evidence that a questionnaire captures clinically relevant aspects of caries risk.

The aim of the present study was to develop and clinically validate a simplified CAMBRA-based caries risk assessment instrument for young adults. The proposed tool was designed to enable rapid risk stratification based exclusively on questionnaire data, without the need for radiographic or laboratory investigations. Its validity was evaluated by assessing internal consistency, temporal stability, and associations between questionnaire-derived risk scores and established clinical indicators of caries experience and activity, with the objective of supporting efficient, risk-based caries prevention in routine preventive dental practice.

## 2. Materials and Methods

Study design: Our study was designed as an observational, cross-sectional investigation aimed at developing and validating a simplified caries risk assessment instrument derived from the CAMBRA conceptual framework. The study comprised two consecutive components: (1) the development of a simplified CAMBRA-based questionnaire and (2) its initial psychometric and clinical validation. In addition, a subsample of participants was reassessed after 14 days to evaluate the temporal stability of the instrument.

The study protocol was conducted in accordance with the principles of the Declaration of Helsinki and received approval from the Ethics Committee of Denta Aur Private Medical Center, Tîrgu Mureș, Romania (protocol no. 11/3 September 2024). All participants provided written informed consent prior to enrolment.

**Study population and recruitment**: Participants were recruited from the general population of Romanian young adults aged 18–25 years, using both academic and non-academic settings. Recruitment sites included university environments as well as community-based locations, allowing the inclusion of individuals both enrolled and not enrolled in formal education at the time of the study. This approach was chosen to reflect the heterogeneity of young adults encountered in routine preventive dental practice and to enhance the external applicability of the proposed instrument.

Participants were invited through direct approach and informational announcements in university-associated and community-based settings. Young adults who presented for the clinical visit completed the questionnaire under supervision immediately prior to the oral examination. Following clinical assessment, three participants were excluded due to the identification of exclusion criteria not declared at recruitment, resulting in a final sample of 246 participants. A formal a priori sample size calculation was not performed; however, the final sample size (*n* = 246) exceeds commonly recommended thresholds for exploratory psychometric validation studies and was therefore considered adequate for the analyses performed. Eligibility criteria. Individuals were included if they met the following criteria: age between 18 and 25 years, presence of natural dentition with at least 20 permanent teeth, ability to complete a self-administered questionnaire in Romanian, and provision of informed consent.

Participants were excluded if they were undergoing active fixed orthodontic treatment, presented systemic conditions with a known major impact on oral health, were under chronic medication with significant xerogenic effects, or were unable to reliably complete the questionnaire. This category referred to systemic conditions known to significantly affect salivary function or oral health status, such as Sjögren’s syndrome, uncontrolled diabetes, previous head and neck radiotherapy, or conditions requiring long-term xerogenic medication. Common systemic conditions without a substantial direct impact on oral health were not considered exclusion criteria. Participants with incomplete clinical or questionnaire data were also excluded from the final analysis.

### 2.1. Development of Simplified CAMBRA-Based Instrument

The caries risk assessment instrument evaluated in this study was newly developed by adapting and simplifying key domains from the CAMBRA framework. Item selection was guided by clinical relevance, feasibility in non-specialized settings, and applicability to young adult populations. Domains requiring radiographic, salivary, or microbiological investigations were intentionally excluded to retain parameters that can be reliably captured through questionnaire-based assessment and applied in routine preventive settings. Item selection was performed by four clinicians with experience in preventive dentistry and caries risk assessment, based on parameters commonly evaluated during preventive dental consultations, with the contribution of an external expert involved in the development of the instrument.

The resulting questionnaire included a reduced number of items addressing biological risk factors, protective factors, and behavioral aspects commonly assessed during preventive dental consultations. The instrument was intentionally designed to be completed within a few minutes and without the need for laboratory tests, salivary analyses, or radiographic investigations, making it suitable for large-scale screening and routine preventive use.

Prior to data collection, the questionnaire underwent a pilot testing phase to ensure clarity, comprehensibility, and feasibility of completion in a clinical setting. Minor wording adjustments were made to improve clarity, but no structural modifications to the questionnaire domains were introduced. Items corresponding to protective factors were coded as present (1) or absent (0) at the time of data collection. During score computation, the cumulative protection score was subtracted from the sum of disease indicators and risk factors, in accordance with the CAMBRA conceptual framework.

The questionnaire used in this study is provided in Supplementary File S1, and the detailed scoring algorithm and risk categorization procedure are provided in Supplementary File S2.

### 2.2. Clinical Oral Examination

All participants underwent a standardized clinical oral examination performed by three calibrated clinicians. Prior to data collection, the examiners underwent a calibration session to standardize the clinical assessment criteria. During this phase, clinical findings were independently evaluated and subsequently discussed to ensure consistent interpretation of DMFT scoring and plaque assessment criteria. Inter-examiner agreement for DMFT and plaque assessment was evaluated during the calibration phase, and a high level of agreement between examiners was achieved before the start of the study. Clinical examinations were performed under standardized lighting conditions, using dental mirrors and air-drying when necessary. The examination focused on the identification of visual clinical indicators relevant to caries risk assessment, including visible dental plaque, oral hygiene status, initial enamel changes, cavitated carious lesions, and selected structural features associated with increased caries susceptibility.

The DMFT index was assessed exclusively through visual clinical examination, without radiographic evaluation. Only clinically cavitated carious lesions were recorded as decayed teeth. Initial non-cavitated enamel lesions, including white spot lesions, were not included in the DMFT score and were analyzed separately as indicators of early disease activity. Missing teeth were recorded as part of the DMFT index only when tooth loss was attributable to dental caries, based on clinical judgment and participant-reported history. Teeth lost for orthodontic, traumatic, or congenital reasons were not considered. Filled teeth were defined as teeth presenting permanent restorations, including crowns, in the absence of clinical signs of secondary caries. Preventive sealants and temporary restorations were not recorded as filled teeth.

This conservative scoring approach was adopted to ensure that caries risk assessment reflects current disease activity rather than historical disease experience alone.

### 2.3. Data Collection Procedure

The questionnaire was completed by participants using a secure online platform in a clinical setting, under investigator supervision, immediately prior to the oral examination. The clinical assessment was subsequently performed independently by calibrated examiners who were blinded to questionnaire responses, to minimize observational bias. For the assessment of test–retest reliability, the questionnaire was re-administered and completed after a 14-day interval by a subsample of 73 participants.

### 2.4. Statistical Analysis

Statistical analyses were performed using IBM SPSS Statistics for Windows, version 26.0 (IBM Corp., Armonk, NY, USA). Internal consistency of the questionnaire was evaluated using Cronbach’s alpha coefficient. Test–retest reliability was assessed using the intraclass correlation coefficient (ICC). Construct validity was explored by examining the relationships between questionnaire-derived risk scores and clinically recorded indicators obtained during oral examination, including DMFT values and the presence of early enamel changes. Differences in risk scores across predefined clinical categories were analyzed using the Kruskal–Wallis test due to the non-normal distribution of the data. This test evaluated overall differences across the predefined caries risk categories, and the reported *p*-values correspond to these global comparisons between groups. Associations between caries risk categories and clinical indicators were examined using non-parametric comparisons across predefined groups. Statistical significance was set at a two-tailed *p* value < 0.05. Exploratory analyses were conducted to assess potential differences in caries risk scores according to participants’ enrolment in formal education at the time of the study, using non-parametric comparative tests as appropriate.

### 2.5. Methodological Considerations

The study was conceived from a pragmatic clinical perspective, aiming to balance methodological rigor with applicability in everyday preventive dentistry. The proposed instrument was not intended to replace comprehensive caries management protocols, but rather to provide a rapid and clinically meaningful tool for caries risk stratification in young adult populations.

## 3. Results

### 3.1. Characteristics of the Study Population

A total of 246 young adults aged between 18 and 25 years were included in the final analysis. The mean age of the participants was 21.4 years (SD 2.1). Females were slightly more represented than males. Most participants were enrolled in formal education at the time of recruitment, while a smaller proportion were not enrolled in formal education Table 1).

### 3.2. Distribution of Caries Risk Scores and Categories

The simplified CAMBRA-based instrument generated a broad distribution of caries risk scores within the study population. Based on the predefined cut-off values, participants were classified into four categories (Table 2). Low caries risk was identified in a minority of participants, whereas the moderate-risk category accounted for the largest proportion of the sample. A substantial number of participants were classified as high risk, while a smaller but clinically relevant group fell into the very high-risk category. Overall, the distribution of risk categories suggested that the instrument was able to differentiate between distinct levels of caries risk in this young adult population.

### 3.3. Reliability of the Instrument

The internal consistency of the questionnaire was acceptable. The Cronbach’s alpha coefficient for the total score was 0.71, indicating adequate coherence between items addressing disease indicators, risk factors, and protective behaviors.

Test–retest reliability was assessed in a subsample of 73 participants who completed the questionnaire twice, with a 14-day interval between administrations. The intraclass correlation coefficient (ICC) for the total risk score was 0.82 (95% CI: 0.74–0.88), indicating good temporal stability of the instrument (Table 3).

### 3.4. Construct Validity

Higher caries risk categories were associated with less favorable clinical findings (Table 4). Median DMFT values increased progressively across caries risk categories, with lower values observed in participants classified as low risk and higher values in those classified as very high risk. Differences between groups were statistically significant.

Initial enamel changes (white spot lesions) were more frequently observed in participants classified as high and very high risk compared to those in the low- and moderate-risk categories. Similarly, visible dental plaque and poor oral hygiene scores were more commonly recorded in the higher-risk groups.

Active cavitated lesions (D > 0) were predominantly identified among participants classified as high or very high risk, supporting the clinical relevance of the proposed risk stratification.

### 3.5. Exploratory Analysis According to Enrolment in Formal Education

Exploratory analyses showed modest differences in caries risk distribution between participants enrolled in formal education and those not enrolled at the time of the study. Participants not enrolled in formal education tended to be more frequently classified in the higher-risk categories. However, the overall pattern of associations between caries risk categories and clinical indicators remained consistent across educational groups.

## 4. Discussion

The present study reports the clinical performance of a simplified CAMBRA-based caries risk assessment instrument when applied to a young adult population in a real preventive care setting. Within this context, the proposed instrument may also function as a practical diagnostic screening tool, supporting early identification of individuals at increased caries risk and facilitating risk-based preventive decision-making in routine clinical practice. The questionnaire showed acceptable internal consistency and good temporal stability, together with consistent associations with clinically recorded indicators of caries experience and activity. These findings support the clinical relevance of questionnaire-based approaches for initial caries risk stratification in routine preventive dental practice, in line with contemporary risk-oriented caries management concepts [7,11,17,18,19,20].

In the present sample, higher questionnaire-derived risk categories corresponded to progressively less favorable clinical findings. Participants classified as high or very high risk exhibited higher DMFT values, a greater prevalence of initial enamel changes, increased levels of visible dental plaque, and a higher frequency of active cavitated lesions compared with those classified as low or moderate risk. The observed gradients across risk categories indicate that the instrument can distinguish between clinically distinct risk profiles, capturing both accumulated caries experience and signs of ongoing disease activity. Similar associations between risk assessment outcomes and clinical indicators have been reported in previous validation studies of CAMBRA-based tools and other caries risk assessment models, supporting the construct validity of the present approach [21,22,23].

The pragmatic design of the proposed tool addresses several practical limitations associated with comprehensive caries risk assessment systems. While full CAMBRA protocols and multifactorial models such as the Cariogram integrate a wide range of clinical, behavioral, and biological parameters, including radiographic findings and salivary or microbiological assessments, their routine application may be limited in busy clinical environments or community-based preventive programs [13,24,25]. Unlike predictive models such as the Cariogram, which aim to estimate future caries incidence or risk, the present cross-sectional study evaluated associations between questionnaire-derived risk categories and current clinical indicators. Longitudinal studies will be required to assess the predictive performance of the proposed instrument. By relying exclusively on questionnaire-based information, the proposed instrument allows rapid initial risk stratification without the need for radiographic assessment, salivary testing, or additional chairside procedures, which may facilitate its integration into routine preventive care. Importantly, the present findings indicate that this simplified approach retains clinical relevance when appropriately validated against objective oral health indicators [11,26,27]. Although biological parameters such as salivary flow, buffering capacity, or microbial load were not included in the simplified instrument, the observed associations between questionnaire-derived risk categories and clinical indicators of caries experience and activity suggest that the instrument is able to distinguish between clinically relevant risk profiles.

Several methodological considerations should be acknowledged when interpreting these results. The cross-sectional design does not permit conclusions regarding the predictive performance of the instrument over time, and longitudinal studies are required to assess its ability to predict caries incidence or progression, as highlighted in previous evaluations of caries risk assessment tools [10,13,28]. In addition, DMFT assessment was based on visual clinical examination without radiographic evaluation, which may have resulted in underdiagnosis of certain interproximal lesions. According to recent expert consensus, caries management in clinical practice should integrate caries risk assessment with lesion activity and be supported by individualized treatment planning and periodic reassessment over time [29]. In addition, although the questionnaire was administered under supervision to reduce missing data and misinterpretation, the use of self-reported information may still introduce recall or reporting bias. This may also include the influence of social desirability bias, whereby participants could overestimate adherence to recommended oral hygiene behaviors or underreport cariogenic dietary habits, particularly when completing the questionnaire in a supervised clinical environment. The distribution of educational status within the sample may also have influenced the observed risk profile; however, the associations between risk categories and clinical indicators remained consistent across groups. In addition, socioeconomic variables such as income, occupation, or parental education level were not systematically collected and may represent potential confounding factors. Nevertheless, questionnaire-based assessments are widely used in preventive and epidemiological research, and their utility is supported when consistent associations with objective clinical indicators are demonstrated [12,30,31,32]. The coherence observed between questionnaire-derived risk categories and multiple clinical parameters in the present study supports the validity of the approach within the investigated population.

Taken together, these findings support the use of the simplified CAMBRA-based questionnaire as an initial screening and decision-support aid in young adults, intended to complement, rather than substitute, comprehensive clinical caries assessment. Its application may be particularly valuable in preventive and community-based settings where rapid risk stratification is required to support efficient, risk-oriented preventive decision-making and allocation of preventive resources [13,17,33,34]. From a practical perspective, the simplified structure of the instrument may also facilitate future digital implementation. Further studies could explore the application of the questionnaire in broader population groups and support the development of web-based platforms that would allow automated scoring and easy access for dental practitioners, potentially facilitating large-scale preventive screening.

## 5. Conclusions

The simplified CAMBRA-based caries risk assessment instrument demonstrated acceptable reliability and clinically meaningful associations with established indicators of caries experience and activity in young adults, supporting its potential use as a diagnostic screening tool in routine preventive dental practice. By enabling rapid risk stratification based exclusively on questionnaire data, the proposed tool may support efficient, risk-based preventive decision-making in routine dental practice. Its use may be particularly valuable in community and population-based settings where comprehensive clinical assessment is not immediately feasible. Further longitudinal studies are warranted to evaluate its predictive performance over time.

## Figures and Tables

**Table 1:** Characteristics of the study population.

Variable	Value
Number of participants, *n*	246
Age, mean ± SD (years)	21.4 ± 2.1
Female sex, *n* (%)	142 (57.7)
Male sex, *n* (%)	104 (42.3)
Enrolled in formal education, *n* (%)	162 (65.9)
Not enrolled in formal education, *n* (%)	84 (34.1)

**Table 2:** Distribution of caries risk categories.

Caries Risk Category	*n* (%)
Low risk	48 (19.5)
Moderate risk	96 (39.0)
High risk	72 (29.3)
Very high risk	30 (12.2)

**Table 3:** Reliability analysis of the simplified CAMBRA-based instrument.

Parameter	Value
Cronbach’s alpha	0.71
Test–retest sample (*n*)	73
ICC (95% CI)	0.82 (0.74–0.88)

**Table 4:** Clinical indicators across caries risk categories.

Indicator	Low Risk	Moderate Risk	High Risk	Very high Risk	*p*-Value
DMFT, median (IQR)	3 (2–4)	5 (4–6)	7 (6–9)	9 (8–12)	<0.001
White spot lesions, *n* (%)	6 (12.5)	20 (20.8)	28 (38.9)	16 (53.3)	<0.01
Visible plaque (score = 2), *n* (%)	4 (8.3)	18 (18.8)	30 (41.7)	18 (60.0)	<0.001
Active caries (D > 0), *n* (%)	2 (4.2)	12 (12.5)	26 (36.1)	18 (60.0)	<0.001

## Data Availability

The data presented in this study are not publicly available due to ethical and privacy restrictions involving human participants. Anonymized data may be made available from the corresponding author upon reasonable request, subject to institutional and ethical approval.

## Supplementary Materials

The following supporting information can be downloaded at: https://www.mdpi.com/article/10.3390/diagnostics16060859/s1, File S1: Simplified CAMBRA-Based Caries Risk Assessment Questionnaire; File S2: Scoring Algorithm and Risk Categorization Procedure.
