# Supplementary material for: A Simplified CAMBRA-Based Diagnostic Caries Risk Assessment Tool for Young Adults: Development and Clinical Validation
Source: Diagnostics (Basel). 2026 Mar 13;16(6):859. doi: 10.3390/diagnostics16060859 (PMC13025520; doi:10.3390/diagnostics16060859)
Supplement: Supplementary file 1 [file diagnostics-16-00859-s001.zip › Supplementary File S1. Simplified CAMBRA-Based Caries Risk Assessment Questionnaire.docx.pdf]

Denta Aur Dental Medicine Clinic

Târgu Mureș, Romania

SIMPLIFIED CAMBRA QUESTIONNAIRE (Ages 18–25)

Instructions: Please tick the option that best describes you over the **past 12 months**.
Completion time: approximately **3–4 minutes**.

1. Disease Indicators

Question	Answer	Score
Have you had new dental caries or new fillings in the past year?	☐ No (0) ☐ Yes (+2)	
Do you have teeth that are sensitive to cold, sweet foods, or chewing?	☐ No (0) ☐ Yes (+2)	
Have you noticed white or brown spots on your teeth?	☐ No (0) ☐ Yes (+1)	

2. Risk Factors

Question	Answer	Score
How often do you eat or drink sugary products between meals?	☐ Rarely (0) ☐ 1–2×/day (+1) ☐ ≥3×/day (+2)	
How often do you brush your teeth with fluoridated toothpaste (1450 ppm F)?	☐ Twice/day (0) ☐ Once/day (+1) ☐ Less often (+2)	
Do you use dental floss or interdental brushes?	☐ Yes (0) ☐ Sometimes (+1) ☐ No (+2)	
Do you drink sugary soft drinks, energy drinks, or sweet iced tea daily?	☐ No (0) ☐ Once/day (+1) ☐ ≥2×/day (+2)	
Do you frequently experience dry mouth?	☐ No (0) ☐ Sometimes (+1) ☐ Often (+2)	
Do you smoke or use vaping/nicotine products?	☐ No (0) ☐ Yes (+1)	
When was your last routine dental check-up?	☐ <1 year (0) ☐ 1–2 years (+1) ☐ >2 years (+2)	

3. Protective Factors

Question	Answer	Score
Do you use a fluoridated mouthwash (e.g., Elmex, Listerine Fluoride)?	☐ Yes (−1) ☐ No (0)	
Have you had professional dental cleaning or prophylaxis in the past year?	☐ Yes (−1) ☐ No (0)	
Do you have pit and fissure sealants on your molars (if known)?	☐ Yes (−1) ☐ No / Not sure (0)	
Have you received a professional fluoride varnish application in the past 6–12 months?	☐ Yes (−1) ☐ No (0)	

Total questionnaire score

(Disease indicators + Risk factors) − Protective factors =

4. Visual Clinical Indicators (Concomitant Validation)

Indicator	Description	Score
Visible dental plaque	0 = absent; 1 = thin marginal plaque; 2 = clearly visible on ≥2 surfaces	
White spot lesions	0 = absent; 1 = present (≥1 smooth surface, chalky opacity)	
Overall oral hygiene	1 = good; 2 = fair; 3 = poor	
Visual DMFT	Number of affected teeth (D + F)	
Unsealed retentive pits and fissures	0 = absent; 1 = present on ≥1 molar; 2 = multiple, plaque-retentive	
Exposed root surfaces	0 = absent; 1 = present on 1–2 teeth; 2 = extensive (≥3 teeth)	

Total visual clinical indicators score:

Rapid Case Report Form (CRF)

ID: ___________
Sex / Age: ______ / ______

Final questionnaire score: ______

Final caries risk:
☐ Low ☐ Moderate ☐ High ☐ Very high

Date: ________________________**Examiner signature:** ________________________
