# Supplementary material for: A Simplified CAMBRA-Based Diagnostic Caries Risk Assessment Tool for Young Adults: Development and Clinical Validation
Source: Diagnostics (Basel). 2026 Mar 13;16(6):859. doi: 10.3390/diagnostics16060859 (PMC13025520; doi:10.3390/diagnostics16060859)
Supplement: Supplementary file 1 [file diagnostics-16-00859-s001.zip › Supplementary File S2. Scoring Algorithm and Risk Categorization Procedure.docx.pdf]

SCORING AND CARIES RISK CATEGORIZATION ALGORITHM

This document describes the scoring procedure and caries risk categorization algorithm used in the study entitled:

“A Simplified CAMBRA-Based Diagnostic Caries Risk Assessment Tool for Young Adults: Development and Clinical Validation”.

1. Questionnaire Scoring

The simplified CAMBRA-based questionnaire generates a total score calculated as follows:

Total score = (Disease indicators + Risk factors) − Protective factors

Disease indicators include self-reported history of caries or restorations, dental sensitivity, and the presence of white or brown enamel spots.

Risk factors include dietary sugar exposure, oral hygiene practices, fluoride use, tobacco or nicotine use, dry mouth, and dental attendance patterns.

Protective factors include professional fluoride exposure, dental prophylaxis, pit and fissure sealants, and the use of fluoridated mouth rinses.

2. Initial Caries Risk Categories

Based on the total questionnaire score, participants are classified into one of four initial caries risk categories:

• 0–1 points: Low risk
• 2–4 points: Moderate risk
• 5–7 points: High risk
• ≥8 points: Very high risk

3. Clinical Adjustment Rules (Override Criteria)

Following questionnaire-based categorization, visual clinical indicators are used to adjust the
final caries risk category where appropriate.

The presence of any of the following findings results in an automatic classification of at least high caries risk:

• Active cavitated carious lesions (D > 0)
• Dental pain related to caries
• White spot lesions indicating active demineralization

The caries risk category is increased by one level under the following conditions:

• Visible dental plaque score = 2 combined with overall oral hygiene score = 3
• Unsealed retentive pits and fissures score = 2
• Exposed root surfaces score = 2

The caries risk category may be reduced by one level when all of the following conditions are met:

• Visible plaque score = 0
• Overall oral hygiene score = 1
• Absence of active carious lesions and white spot lesions

Risk reduction is not permitted below the low-risk category.

4. Final Risk Classification

The final caries risk category is recorded after applying all clinical adjustment rules and is used for analytical purposes and clinical interpretation within the study.
